# Echinacoside, an Inestimable Natural Product in Treatment of Neurological and other Disorders

**DOI:** 10.3390/molecules23051213

**Published:** 2018-05-18

**Authors:** Jingjing Liu, Lingling Yang, Yanhong Dong, Bo Zhang, Xueqin Ma

**Affiliations:** 1 Department of Pharmaceutical Analysis, School of Pharmacy, Ningxia Medical University, 1160 Shenli Street, Yinchuan 750004, China; 15709604029@163.com (J.L.); 15926361499@163.com (L.Y.); dyh794200808@163.com (Y.D.); zhangbobo0624@163.com (B.Z.); 2 Key Laboratory of Hui Ethnic Medicine Modernization, Ministry of Education, Ningxia Medical University, 1160 Shenli Street, Yinchuan 750004, China

**Keywords:** echinacoside, preparation, pharmacokinetics, Parkinson’s disease, Alzheimer’s disease

## Abstract

Echinacoside (ECH), a natural phenylethanoid glycoside, was first isolated from *Echinacea angustifolia* DC. (Compositae) sixty years ago. It was found to possess numerous pharmacologically beneficial activities for human health, especially the neuroprotective and cardiovascular effects. Although ECH showed promising potential for treatment of Parkinson’s and Alzheimer’s diseases, some important issues arose. These included the identification of active metabolites as having poor bioavailability in prototype form, the definite molecular signal pathways or targets of ECH with the above effects, and limited reliable clinical trials. Thus, it remains unresolved as to whether scientific research can reasonably make use of this natural compound. A systematic summary and knowledge of future prospects are necessary to facilitate further studies for this natural product. The present review generalizes and analyzes the current knowledge on ECH, including its broad distribution, different preparation technologies, poor pharmacokinetics and kinds of therapeutic uses, and the future perspectives of its potential application.

## 1. Introduction—Treasure from the Garden: The Discovery and Distribution of ECH

Phenylethanoid glycosides (PhGs) are naturally occurring water-soluble compounds that are widely distributed in the plant kingdom, and most of which are isolated from garden plants and medicinal herbs. Structurally, these compounds are characterized by cinnamic acid and hydroxyl phenyl ethyl moieties that are attached to a β-glucopyranose (apiose, galactose, rhamnose, xylose, etc.) via a glycosidic bond. In recent years, interest has been growing in using PhGs [1] as their potential in the prevention and treatment of various human diseases and disorders. 

**Figure 1:**
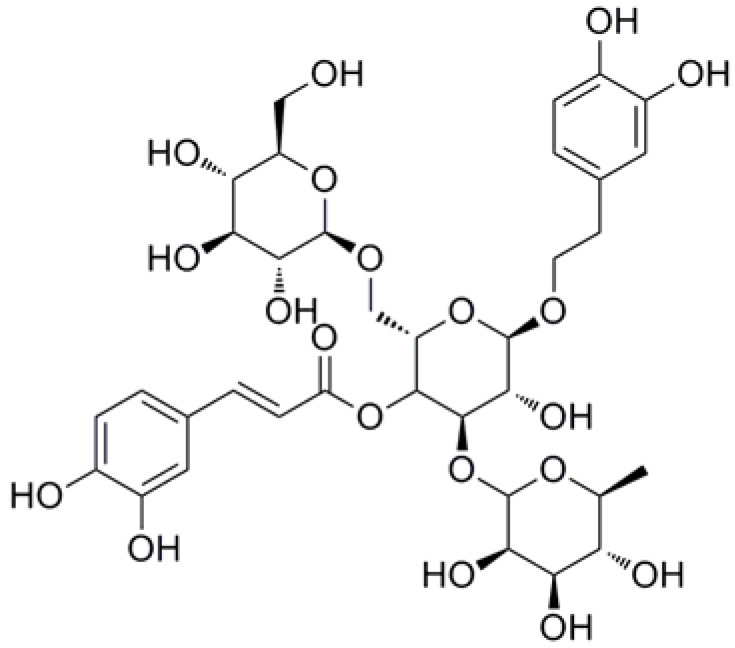
Chemical structure of Echinacoside (ECH) (Glu-Glu-Rha).

**Figure 2:**
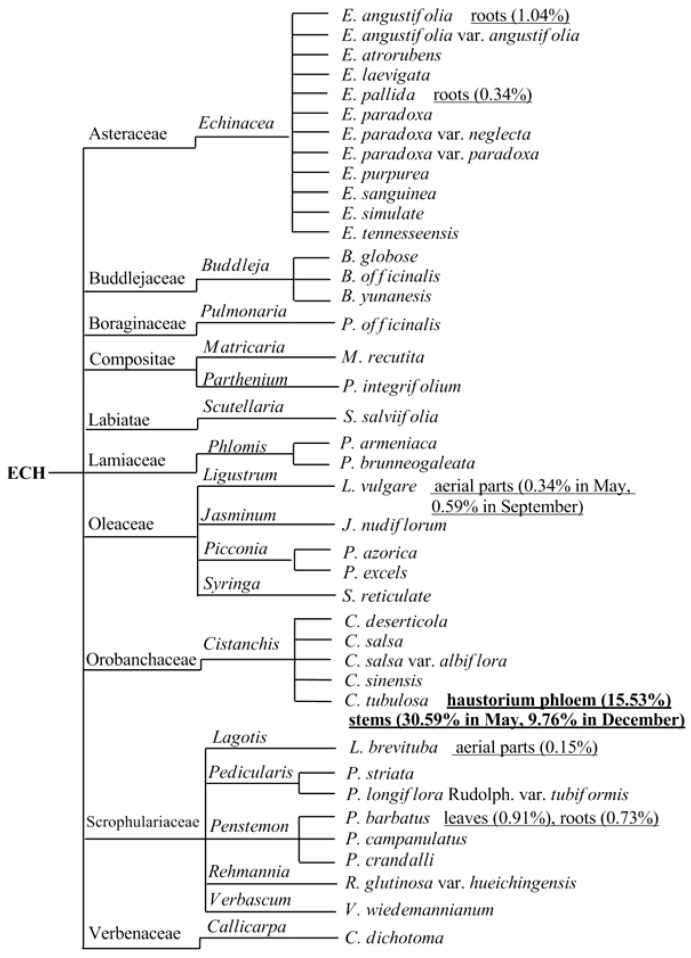
The broad distribution and discovery of ECH in the plant kingdom.

Echinacoside (ECH, Figure 1), a natural PhG, was first isolated from *Echinacea angustifolia* DC. (Compositae), a garden plant sixty years ago, [2] and subsequently prepared from the species of *Cistanches* [3] as well as the aerial part of landscape herb, *Penstemon crandallii* A. Nels. (Scrophulariaceae) [4], whole plants of *Pedicularis striata* Pall. [5] and now successively found in 40 plant species [6,7,8,9,10,11,12] belonging to 18 genus and 10 families (Figure 2). To date, the species of genus *Cistanches* (Orobanchaceae) and *Echinacea* (Asteraceae) were the main natural plant sources for the preparation of ECH. 

ECH was found in both underground and aboveground parts of medicinal herbs but with widely varying levels of content (Figure 2), including different stages of plant growth [8,13], different parts of the same plant [14], and vice versa, the same parts of different plants [15,16]. Until now, the highest content of ECH was found in haustorium phloem of *Cistanches tubulosa* which was reached almost 15.5% [17], thus could be a good resource for the isolation of pure ECH.

## 2. Preparation of ECH

**Figure 3:**
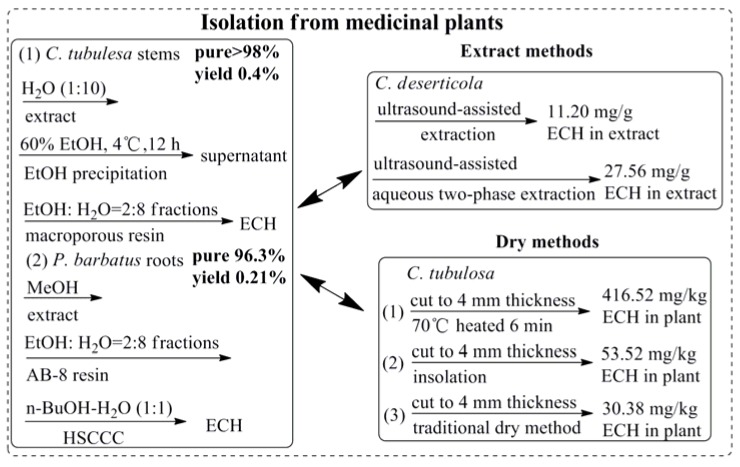

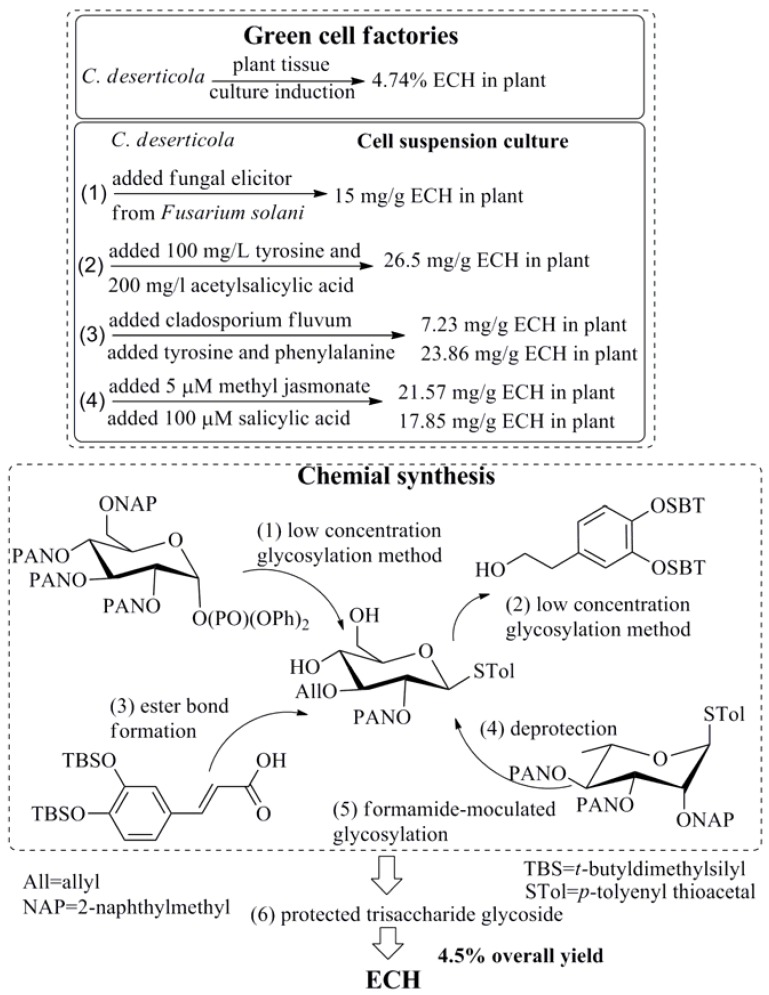
Different methods of ECH obtaining.

As PhG compounds exhibited significant activities in the prevention and treatment of various human diseases and disorders, it was important to develop sustainable methods to produce sufficient quantities of ECH for pharmaceutical applications. Firstly and also usually, like other natural bioactive compounds, high purity of ECH was obtained from medicinal plants by using classic isolation methods and semi-preparative liquid chromatography (LC) [18] or high-speed counter-current chromatography methods [14] as Figure 3 shown, and the yield of ECH was usually between 0.2%~0.4%. An efficient ultrasound-assisted aqueous two-phase extraction process for preparation of ECH from *Cistanche deserticola* enhanced the content of ECH in the extracts (27.56 mg/g) which was 2.46-fold higher than the amounts obtained in ultrasound-assisted extraction [19]. Interestingly, it was found the content of ECH in medicinal herbs was significantly influenced by the factors of preparation processing [20], including the slice thickness, drying temperature, and the time for inactivation of the enzyme [21]. Therefore, it was worth noting that ECH was demonstrated to be highly susceptible to “enzymic” degradation and oxidation in hydroalcoholic solutions during the extraction process, and ECH in biosamples was susceptible to degradation at a higher temperature during the whole process, thus the operation must be carried out carefully at a lower temperature. Secondly, besides the above classic isolation method, plant cell/tissue culture, called “green cell factories”, has become increasingly attractive as a cost-effective alternative to classical approaches for the sustainable mass production of plant-derived molecules [22]. Several published data demonstrated an increased accumulation of ECH in both plant tissue culture [23] and cell suspension culture [24] of *Cistanche deserticola*, and some revulsant including tyrosine, phenylalanine, cladosporium fulvum, methyl jasmonate and salicylic acid were found could promote the accumulation of ECH [25,26,27]. Thirdly, as an inestimable natural product which possesses a broad spectrum of beneficial activities, the chemical synthesis of ECH is needed to satisfy its comprehensive application. A group from National Taiwan University has completed the total synthesis of ECH from building blocks over 7 steps with yield was 4.5% [28] which was showed in Figure 3.

## 3. Pharmacokinetics and Strategy

Generally, the systemic effects of natural PhGs mainly depend on their bioavailabilities through the gastrointestinal barrier. However, both in vitro and in vivo experimental data appeared to reflect their pitiful fates in the gut, including relatively poor bioavailabilities and rapid rates of excretion [29]. Dozens of studies have shown that the content of PhGs in plant within μg, following ingestion, they appeared in the circulation as phase II metabolites, and their plasma levels rarely exceed nM concentrations [30]. Animal study confirmed that the absolute bioavailability of ECH was only 0.83% [31], as Table 1 shown, the absorption and elimination of ECH was extremely fast in rats and the serum concentration was very low, and ECH could not be identified in any human plasma sample at any time after ECH tablet ingestion [32]. The serum concentration–time curves for intragastric and intravenous administration were fitted to one-compartment model and two-compartment model, respectively. The metabolites of ECH in rat feces were identified as acteoside, decaffeoylacteoside, lugrandosie and 3,4-dihydrophenyl ethanol after oral administration [33], and a portion of ECH was transformed into acteoside [34]. Now, to predict the absorption of orally administrated drugs, Caco-2 monolayer is widely used as a model of the human intestinal mucosa. It was shown that ECH permeated poorly through the Caco-2 monolayers although one potential metabolite, cinnamic acid, diffused readily with an apparent permeability of 1 × 10^−4^ cm/s. The data implied ECH was not likely to cross the normal intestinal barrier [35,36,37], but it can through the blood-brain barrier in permanent middle cerebral artery occlusion (MCAO) rats [38]. Furthermore, a recent study estimated the dynamic pharmacokinetic of ECH between Parkinson’s disease rat and normal rats showed that the plasma concentrations of ECH in Parkinson’s disease rats were higher than that in the normal rats after oral administration. The reasons why the elimination rate of ECH slowed down in Parkinson’s disease rats may be as follows: ECH is partially hydrolyzed to aglycone in the body and in the state of pathophysiology, a low activity of certain enzymes induced by 6-hydroxydopamine (6-OHDA) damage might lead to the decreased clearance rate and increased retention time of ECH. Even if ECH was mainly excreted in the urine, the decreased blood circulation of kidney induced by 6-OHDA damage might play an important role in the decreased elimination rate and increased retention time of ECH [39]. 

## 4. Pharmacological Properties and Underlying Mechanisms

**Figure 4:**
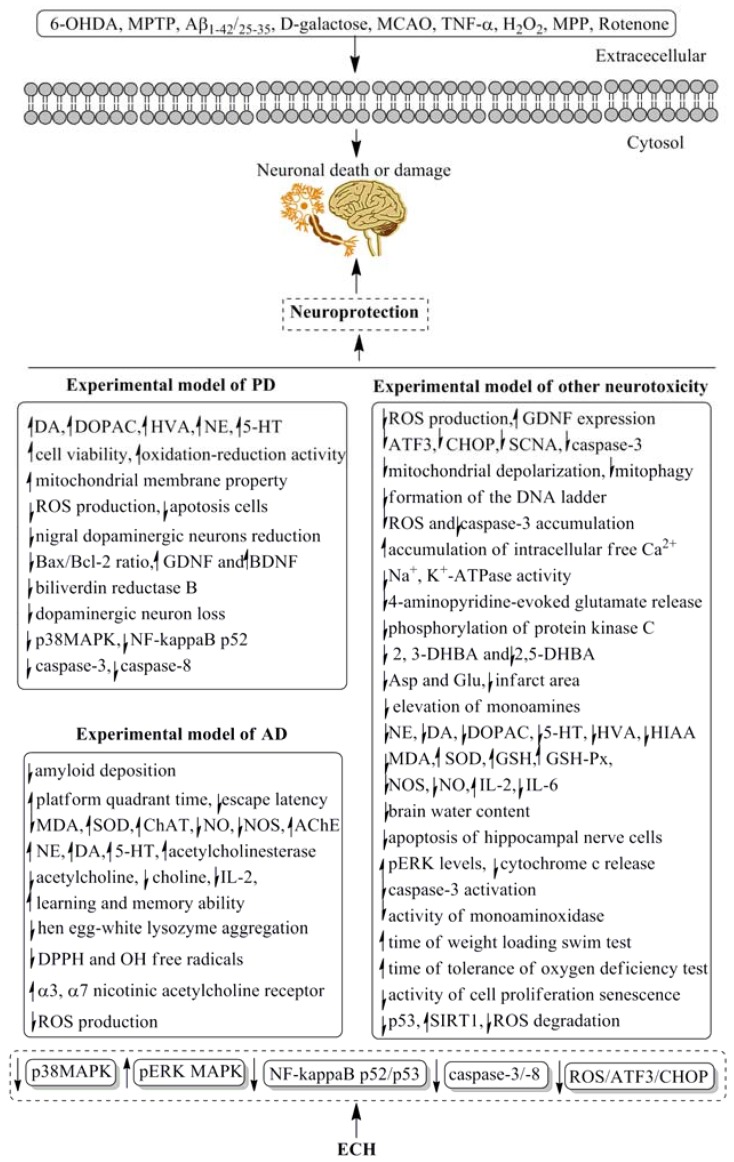
The underlying mechanism of neuroprotective of ECH. AD, Alzheimer’s disease; PD, Parkinson’s disease.

ECH was proved possessing kinds of pharmacological activities since it was found sixty years ago, the data of this review were mainly gathered by consulting the database of PubMed, Springer, Elsevier, and Scholar in the last 30 years. Among the broad range of therapeutic applications of ECH, its neuroprotective bioactive has attracted the more attention of pharmaceutical scientists than the others. Dozens of reports have discovered that ECH was effective in Parkinson’s and Alzheimer’s diseases by using both animal experiments and cell lines tests, which were shown in Table 2. Besides the neuroprotective action, the cardioactive property, anti-inflammatory activity, antioxidant and anti-osteoporotic activities as well as other pharmacological potentials of ECH were presented in Table 3, Table 4, Table 5, Table 6 and Table 7, respectively. The data of Table 2 and Figure 4 showed that ECH could prevent the progress of neurodegeneration in Parkinson’s and Alzheimer’s diseases. Several Parkinson’s or Alzheimer’s animal models induced by 6-OHDA, 1-methyl-4-phenyl-1,2,3,6-tetrahydropyridine (MPTP), d-galactose and β-amyloid Aβ-(25–35) as well as cerebral ischemia rats were used to estimate the neuroprotective effects of ECH. PC12 and neuroblastoma SH-SY5Y cell lines were employed to discover the related mechanisms, which were related to the mitogen-activated protein kinase (MAPK), NF-kappa B, caspase 3 and 8 as well as reactive oxygen species (ROS)/activating transcription factor 3 (ATF3)/C/EBP-homologous protein (CHOP) pathways, as Figure 4 showed. However, most of the above data were obtained by using cells or animals, the reliable clinical trials were limited, large-scale evidence-based human clinical trials with specific neuroprotective therapeutic settings are necessary. The same problems have been found by applying other evaluation methods of the different pharmacological properties. 

One of the traditional uses of *Cistanche deserticola* was for treatment of irritable bowel syndrome disease, and ECH was the main bioactive ingredients in this herbal responsible for the activity. To date, dozens of in vivo studies demonstrated the anti-inflammatory property of ECH, the data of Table 4 showed that ECH could suppress the acute colitis in mice induced by dextran sulphate sodium [88], attenuate acute hepatotoxicity in rats induced by d-galactosamine/lipopolysaccharide [89] and carbon tetrachloride (CCl_4_) [90], increase hyaluronan levels and decrease wound contraction for wound healing, modulate inflammatory markers including transforming growth factor (TGF)-β1, NO, alanine aminotransferase (ALT), myeloperoxidase, inflammatory cytokines, and etc.; however, the molecular mechanisms of the anti-inflammatory of ECH were limited, which were only related to the expressions of TGF-β1, capase-3 and TNF-α.

It was worth mentioning that, to date, numerous in vitro and in vivo studies have demonstrated the strong antioxidant property of ECH (Table 5). In DPPH assay, the EC_50_ of ECH was 6.6 μM which was 9.5-fold than Trolox; on xanthine/xanthine oxidase generated superoxide anion radical test, the IC_50_ of ECH was 2.74 μM than tocopherol, and etc. [98,99,100,101]. In vivo experiments, ECH could prompt the ability of anti-oxication, anti-fatigue and anti-stress in vascular dementia rats or subacute aging mice model, and the indirect antioxidant activities of ECH due to the induction or/and activation of major endogenous antioxidant enzymes and inactivation of pro-oxidant enzymes. In addition, the molecular mechanisms of this activity showed that ECH reduced nuclear protein levels of transcription regulator protein BACH1, enhanced heme oxygenase 1 mRNA levels, down-regulated expression of p53, up-regulated the SIRT1 [102]. Furthermore, the structure-activity relationship of antioxidant property of ECH was also estimated. It was believed that the inhibitory oxidative hemolysis activity of ECH was related to the number of phenolic hydroxy groups. ECH, possessing four phenolic hydroxy groups, exhibited stronger antioxidant activities than cistanoside D possessing only two phenolic hydroxy groups, and compound permethylacteoside with no phenolic hydroxy group inhibited oxidative hemolysis weakly [103,104]. 

*Cistanche deserticola* is a traditional Chinese medicine (TCM) called “Desert ginseng” in China owing to its excellent medical functions and nourishing effect. According to the theory of TCM, *Cistanche deserticola* can supplement the kidney, and kidney stores essence and the essence can transform into bone marrow to nourish the bones, which means *Cistanche deserticola* could promote the formation of the bone [112]. As ECH is the main constituent of *Cistanche deserticola*, thus maybe possesses anti-osteoporotic property. And in the anti-osteoporotic tests as Table 6 shown, ECH exhibited anti-osteoporotic effect on the promotion of bone formation and suppression of bone resorption [113], and the molecular targets of ECH were also discovered that it could increase the osteoprotegerin (OPG) level and decrease the receptor activator for nuclear factor-κB Ligand (RANKL) level [114] as well as promoted the phosphorylation of ERK1/2 to activate MAPK/ERK pathway [115,116]; however, the dosage of ECH were so high that even reached at 270 mg/kg body weight/day, which made some difficult in the future clinical trials and enhanced the medicinal costs. Since the results of the report showed the dosage of 30 mg/kg body weight/day of ECH was also effective in ovariectomized (OVX) rats, a proper dosage of ECH in future treatment of osteoporosis disease should be selected with more tests. 

Besides the above significant bioactive, ECH also proved to possess additional antidiabetic effect, antiviral activity, anti-hepatic fibrosis effect, anti-tumor property, testis and sperm injury protect activities as Table 7 shown.

## 5. Discussion

ECH, a natural PhGs compound has been isolated from dozens of medicinal or horticultural plants, exhibited highly positive activities in treatment of nervous, cardiovascular and bone disorders, especially for the prevention and treatment of a variety of nervous system disorders including Parkinson’s and Alzheimer’s diseases. Given the above potential in pharmaceutical applications, the preparations of ECH including the classic isolation from herbs, plant cell/tissue culture and even the chemical synthesis have attracted the interests of plenty pharmaceutical scientists. This review has presented the discovery of ECH including its distribution in the plant kingdom, and the preparation of ECH including the methods of classic isolation from medicinal plants, “green cell factories” of plant cell/tissue culture and chemical synthesis, and the pharmacokinetics data of ECH was also posted for further medicinal uses. Then, the most important section of this paper, the remarkable pharmacological properties of ECH were elucidated, including neuroprotective activity, cardioactive property, anti-osteoporotic effect, anti-inflammatory and antioxidant activities. However, there were four important questions should be pay attention before ECH was used for clinical applications: first, both in vivo and in vitro experiments of ECH reflected its dissatisfied pharmacokinetic property. Concerning the in vivo experiments, ECH exhibited a pitiful fates in the gut, including relatively poor bioavailability [31] (the absolute bioavailability was only 0.83%) and rapid rates of metabolism and excretion, following ingestion, ECH appeared in the circulation as phase II metabolites, and its plasma levels rarely exceed nM concentrations [30]; and in vitro tests showed that ECH permeated poorly through the Caco-2 monolayers, which implied that ECH was not likely to cross the intestinal and blood brain barriers, thus appeared failed to explain its neuroprotective activity and other bioactive [35]. However, it was reported that in neuronal cells and non-neuronal cells which were exposed to rotenone, ECH was able to cross the blood-brain barrier freely [74]. Second, it should be noted that several of the activities especially the anti-osteoporotic one occurred at relatively high concentrations of ECH (30–270 mg/kg/day, orally for 12 weeks) [113], and in the antidiabetic experiments [87], the dose of ECH even reached at 500 mg/kg p.o. And others occurred at normal concentrations about 5 to 50 mg/kg. Third, the underlying molecular mechanisms of ECH with neuroprotective and cardiovascular properties, anti-osteoporotic, and anti-inflammatory activities have not been elucidated in detail. Although some common molecular signal pathways and several distinct targets have been disclosed, the responses of molecular targets to ECH with the above effects remain unclear. Forth, sixty years after the discovery of ECH, to date, very little efforts are done for its clinical trials and the safety and toxicity tests, and reliable clinical data describing the health effects of ECH are limited. Therefore, the in vivo animal studies of ECH should be considered with caution and more clinical trial on its efficacy and safety should be performed.

## 6. Conclusions

In summary, ECH was an inestimable natural product that exhibited highly positive activities in nervous and cardiovascular system disorders as well as bone disease from the wealth of laboratory data, and thus was believed have a promising potential in the treatment of Parkinson’s and Alzheimer’s diseases, atherosclerosis, osteoporosis, acute colitis, wound injury, and hepatitis. The excellent antioxidant property, which was 9.5-fold greater than Trolox, also implied an ideal application of ECH in the future clinical trials. However, at present, despite the wealth of experimental data that was available describing the potent pharmacological effects of ECH, many issues remain unresolved with respect to effective clinical applications. First, the low bioavailability and extremely fast metabolism of ECH in animals reflected its dissatisfied pharmacokinetic property in the future clinical application. The rapid rates of metabolism and excretion of ECH was due to the multiple metabolic pathways that were involved to eliminate plant-derived toxins, thus further intensive studies are required to confirm the clinical potential of ECH, thereby enabling its acceptance as a therapeutic agent. Another important issue was that, although the present review findings provided a sound basis to confirm that ECH is a potential candidate for intervention in neurodegenerative diseases such as Alzheimer’s and Pakinson’s disorders, the molecular signal pathways, especially the molecular targets to ECH with the above effects, remain unclear. Thus, further studies are needed to interpret the directed molecular mechanisms. The last issue is also the most important problem, implying that large-scale evidence-based human studies with specific therapeutic settings are necessary. Although plenty of laboratory data shed light on the protection of ECH against dozens of diseases, the reliable clinical data are limited. More clinical trials on the safety and drugability of ECH are needed.

## Figures and Tables

**Table 1:** Pharmacokinetics of ECH with different models.

Dose/Model	Pharmacokinetics Parameters including Metabolites	Ref.
100 mg/kg p.o./rats	*T*_max_ = 15.0 min, *C*_max_ = 612.2 ± 320.4 ng/mL, *T*_1/2_ = 74.4 min, *C*_6h_ = 36.3 ng/mL, fit to one-compartment model, absolute bioavailability was 0.83%.	[31,40]
5 mg/kg i.p./rats	*T*_1/2α_ = 12.4 min, *T*_1/2β_ = 41.0 min, *C*_2min_ = 15598.8 ng/mL, *C*_4h_ = 143.6 ng/mL, fit to two-compartment model.	[31,40]
100 mg/kg p.o./rats	Rat feces: acteoside, decaffeoylacteoside, lugrandosie, 3,4-dihydrophenyl ethanol.	[33]
3 g/kg of total PhGs p.o./rats	PhGs are mainly metabolized in large intestine, and the content of ECH fell from 48% to 16%, a portion of which was transformed into acteoside.	[34]
ECH tablet manufactured from ethanolic liquid extracts of *E. angustifolia* and *E. purpurea* p.o./9 healthy volunteers	ECH could not be identified in any human plasma sample at any time.	[32,36]
20 mg/kg p.o./Parkinson’s disease and normal rats	*T*_1/2_ = 73.9 min and *C*_max_ = 403.6 ± 52.3 ng/mL in Parkinson’s disease rats, *T*_1/2_ = 121.6 min and *C*_max_ = 365.2 ± 46.4 ng/mL in normal rats.	[39]
50 mg/kg p.o./MCAO rats	*C*_5min_ = 29.83 ng/mL, *C*_15min_ = 31.28 ng/mL, *C*_30min_ = 40.21 ng/mL, *C*_45min_ = 26.49 ng/mL, *C*_60min_ = 21.20 ng/mL, *C*_90min_ = 14.04 ng/mL, *C*_120min_ blow LOD.	[38]
8.4 ± 1.6 μg/mL/Caco-2 monolayers	Permeated poorly, 0% was uptake at 90 min, and the apparent permeability was zero.	[35,36]
200 μM/Caco-2 monolayers	Passive diffusion, apparent permeability was nearly 10^−7^ cm/s.	[37]

MCAO, middle cerebral artery occlusion; LOD, limit of detection; p.o., intragastric administration; i.p., intraperitoneal injection.

**Table 2:** Neuroprotective activity of ECH in selected models.

Models	Dosage	Mechanism	Ref.
6-OHDA-induced Parkinson’s disease in rats	10, 20 and 40 mg/kg for 4 weeks, i.p.	Increased the striatal and hippocampus extracellular fluid of DA, DOPAC, HVA, NE, and 5-HT levels.	[41]
6-OHDA-induced Parkinson’s disease in rats	3, 5 and 7 mg/kg for 7 days, i.p.	Prevented the decreased of the striatal extracellular levels of DA, DOPAC and HVA.	[42]
6-OHDA-induced neurotoxicity in rats	3, 5 and 7 mg/kg for 7 days, i.p.	Prevented the decreased of the extracellular levels of DA, DOPAC and HVA, elevated the concentrations of DA, DOPAC and HVA in the right striatum of awake, freely-moving rats.	[43]
6-OHDA-induced neurotoxicity in PC12 cells	0.1, 1 and 10 μM	Significantly enhanced cell viability, oxidation-reduction activity and mitochondrial membrane potential, reduced ROS production, as well as inhibited mitochondria-mediated apoptosis.	[44]
MPTP-induced neurotoxicity in mice	30 mg/kg for 14 days, p.o.	Suppressed the reduction of nigral dopaminergic neurons, striatal fibers, DA and DA transporter, prevented the apoptosis cells and Bax/Bcl-2 ratio of mRNA and protein, increased the expression level of GDNF and BDNF mRNA and protein, and improved the gait disorder.	[45]
MPTP-induced Parkinson’s disease in C57BL/6 mice	20 mg/kg for 14 days, p.o.	Protected the C57BL/6 mice against MPTP-induced behavioral default, increased the number of spontaneous movement and latent period of mice on the rotating rod., and decreased the level of protein biliverdin reductase B.	[46]
MPTP-induced Parkinson’s disease in C57BL/6 mice	30 mg/kg for 14 days, p.o.	Suppressed the dopaminergic neuron loss, maintained dopamine and dopamine metabolite content, inhibited the activation of microglia and astrocytes in the substantia nigra; downregulated the level of p38MAPK and the NF-kappaB p52 subunit.	[47]
MPTP-induced Parkinson’s disease in mice	5 and 20 mg/kg for 15 days, p.o.	Reduced behavioral deficits and cell death, increased striatal DA, DA metabolite levels and tyrosine hydroxylase expression; reduced caspase-3 and caspase-8 activation in MPP-induced apoptosis in cerebellar granule neurons.	[48]
MPTP-induced neurotoxicity in SH-SY5Y cells	10, 20 and 40 μg/mL	Improved cell survival, suppressed the generation of ROS and the expression of apoptotic genes (ATF3, CHOP, and SCNA), and decreased the caspase-3 activity in a dose-dependent manner; restored the GDNF expression, improved dopaminergic neuron survival and protected these neurons against apoptosis; protected apoptosis through ROS/ATF3/CHOP pathway.	[49]
Aβ-(1–42)-induced Alzheimer’s disease in rats	25 and 50 mg/kg for 15 days, p.o.	Ameliorated the cognitive deficits, decreased amyloid deposition, reversed cholinergic and hippocampal dopaminergic dysfunction.	[50]
d-galactose coupled with Aβ-(25–35)-induced Alzheimer’s disease in rats	10, 20 and 40 mg/kg for 4 weeks, i.p.	Extended the platform quadrant time, shortened the escape latency, alleviated the learning and memory impairment, and improved the concentrations of NE, DA, 5-HT in the hippocampus and cerebral cortex.	[51]
Occluding the bilateral common carotid arteries induced Vascular dementia in rats	15, 30 and 45 mg/kg for 4 weeks, i.p.	Decreased the content of MDA and increased the activities of SOD, ChAT, and AChE in the hippocampus and cerebral cortex.	[52]
d-galactose coupled with Aβ-(25–35)-induced Alzheimer’s disease in rats	10, 20 and 40 mg/kg for 4 weeks, i.p.	Decreased the content of MDA, increased the activity of SOD, reduced the release of NO and NOS in the hippocampus and cortex brain tissue.	[53]
Rapid aging dementia in sam-p/8 mice	50 mg/kg for 30 days, p.o.	Increased the learning and memory ability, reduced the levels of acetylcholine and IL-2, increased the total anti-oxidative ability.	[54]
Permanent bilateral common carotid artery occlusion induced Vascular dementia in rats	15, 30 and 45 mg/kg for 4 weeks, i.p.	Increased the content of acetylcholine, enhanced the activity of acetylcholinesterase, reduced the content of choline extracellular of hippocampus and striatum.	[55]
Aβ-(25–35)-induced neurotoxicity in PC12 cells	100, 300 and 500 μM	Inhibited hen egg-white lysozyme aggregation occurred in different fiber-forming stages, scavenged the DPPH and OH free radicals, increased viability of PC12 cell line, and suppressed the increase in intracellular ROS.	[56]
Aβ-(25–35)-induced neurotoxicity in SH-SY5Y cells	80 μM	Stimulated the increase of α3 and α7 nicotinic acetylcholine receptor subunit proteins and cell viability.	[57]
MPP induced neurotoxicity in SH-SY5Y cells	5, 10 and 20 μg/l	Suppressed the mitochondrial depolarization, mitophagy and cell apoptosis, exhibited protective effects on mitochondrial function and cell apoptosis.	[58]
TNF-α induced neurotoxicity in SHSY5Y cells	1, 10 and 100 μg/mL	Reduced formation of the DNA ladder, prevented the accumulation of ROS and caspase-3, reconverted the potential of mitochondarial membrane, and decreased the percentage of apoptosis/necrosis neurons.	[59]
TNF-α induced neurotoxicity in SHSY5Y cells	1, 10 and 100 μg/mL	Prevented the accumulation of ROS, maintained the function of mitochondria, inhibited the activity of caspase-3 activity and increased the expression of the antiapoptotic protein Bcl2.	[60]
H_2_O_2_ induced neurotoxicity in SH-SY5Y cells	50 μM	Protected SH-SY5Y cells against H_2_O_2_ induced oxidative injury.	[61]
H_2_O_2_ induced neurotoxicity in PC12 cells	5 and 10 μg/mL	Increased cell viability, decreased the apoptotic ratio, inhibited the formation of ROS and accumulation of intracellular free Ca^2+^, elevated the mitochondrial membrane potential in H_2_O_2_ induced PC12 cells, downregulated Bax protein expression and upregulated Bcl-2 protein expression, and prevented an H_2_O_2_ induced increase of the Bax/Bcl-2 ratio.	[62]
H_2_O_2_ induced neurotoxicity in PC12 cells	10 μM	Increased cell viability and decreased the necrotic ratio, inhibited the formation of NO, down-regulated p65 and iNOS mRNA expressions.	[63]
H_2_O_2_ induced neurotoxicity in PC12 cells	5 and 10 μg/mL	Increased cell viability and Na^+^, K^+^-ATPase activities as well as mitochondrial membrane bioactive, down-regulated the expressions of p53 mRNA and up-regulated the expressions of Bcl-2 mRNA.	[64]
H_2_O_2_ induced neurotoxicity in PC12 cells	1, 5, 10, 30, and 50 μM	Inhibited Ca^2+^ dependent 4-aminopyridine-evoked glutamate release, reduced the 4-aminopyridine-evoked increase in cytoplasmic free Ca^2+^ concentration, decreased the phosphorylation of protein kinase C.	[65]
Permanent MCAO-induced neurotoxicity in rats	15 and 30 mg/kg for 7 days, i.p.	Prevented the elevation of 2, 3-DHBA and 2,5-DHBA.	[66]
Permanent MCAO-induced neurotoxicity in rats	20 and 40 mg/kg for 7 days, i.p.	Decreased the levels of Asp and Glu, reduced the infarct area.	[67]
Permanent MCAO-induced neurotoxicity in rats	15 and 30 mg/kg for 7 days, i.p.	Prevented the elevation of monoamines, NE, DA, DOPAC, 5-HT and HIAA.	[68]
Permanent MCAO-induced neurotoxicity in rats	15 and 30 mg/kg for 7 days, i.p.	Decreased the content of MDA, and increased the activities of SOD and GSH.	[69]
Permanent MCAO-induced neurotoxicity in rats	25 and 50 mg/kg for 7 days, i.p.	Improved neurological deficit, reduced brain water content and the apoptosis of hippocampal nerve cells.	[70]
Permanent MCAO-induced neurotoxicity in rats	15 and 30 mg/kg for 7 days, i.p.	Attenuated the increased of NE, DA, DOPAC, 5-HT and HIAA.	[71]
Permanent MCAO-induced neurotoxicity in rats	15 and 30 mg/kg for 7 days, i.p.	Prevented the extracellular levels of NE, DA, DOPAC, HIAA, HVA and 5-HT.	[72]
Rotenone-induced Parkinson’s disease in rats	20, 40 and 80 mg/kg for 4 weeks, i.p.	Suppressed the neurological disability and the loss of dopaminergic neurons in substantia nigra, increased DA concentrations in striatum, no effect on liver and kidney damage.	[73]
Rotenone-induced injury in SHSY5Y, Hela and HEK293T cells	5, 10 and 20 μg/mL	Protected cells over-expressed with TrkA or TrkB against rotenone injury, elevated the pERK levels and inhibited cytochrome c release and caspase-3 activation.	[74]
Permanent MCAO-induced neurotoxicity in rats	10, 20 and 40 mg/kg for 4 weeks, i.p.	Increased the content of GSH and activity of GSH-Px, decreased the activity of NOS; arranged the rat tissue structure of hippocampal CAI area in order.	[75]
d-galactose induced subacute aging in mice	50 mg/kg for 6 weeks, p.o.	Scavenged the free radicals of OH, O_2_ and L, repaired the damages, enhanced the activities of GSH-PX and SOD, reduced the content of MDA, and decreased the activity of monoaminoxidase thus delay the aging process.	[76]
d-galactose induced subacute aging in mice	20, 40 and 60 mg/kg for 8 weeks, p.o.	Reduced the content of IL-6 and MDA, increased the content of IL-2 and NO, enhanced the immune function and activity of SOD in brain tissue.	[77]
d-galactose induced subacute aging in mice.	20, 40 and 60 mg/kg for 8 weeks, p.o.	Increased the content of IL-2, reduced the content of IL-6, MDA and mitochondrial DNA, improved phagocytosis of peritoneal macrophages and transformation of spleen lymphocytes.	[78]
d-galactose induced subacute aging in mice.	10, 20 and 40 mg/kg for 8 weeks, p.o.	Prolonged the time of weight loading swim test and tolerance of oxygen deficiency test, increased the activity of SOD and the level of IL-2, reduced the content of MDA.	[79]
Replicative induced senescence and H_2_O_2_ induced neurotoxicity in MRC-5 cells	20, 50 and 100 μM	Down-regulated the expression of p53 and up-regulated the expression level of SIRT1.	[80]
Replicative induced senescence and H_2_O_2_ induced neurotoxicity in MRC-5 cells	1, 20, 50 and 100 μM	Retarded the activity of cell proliferation senescence, triggered cells in the G1 phase to enter the S phase and G2 phase, improved the ROS degradation, and protected cells from DNA damage.	[81]

DA, dopamine; DOPAC, 3,4-dihydroxyphenyl acetic acid; HVA, homovanillic acid; NE, norepinephrine; 5-HT, 5-hydroxytryptamine; GDNF, glial cell line-derived neurotrophic factor; BDNF, brain-derived neurotrophic factor; MDA, malondialdehyde; SOD, superoxide dismutase; ChAT, choline acetyltransferaxe; NO, nitric oxide; NOS, nitric oxide synthase; AchE, acetylcholinesterase; DPPH, 2,2-diphenyl-1-picrylhydrazylhydrate; SCNA, synuclein alpha; DHBA, dihydroxybenzoic acid; Asp, aspartic acid; Glu, glutamic acid; HIAA, hydroxyindoleacetic acid; GSH, glutathione; GSH-Px, glutathione peroxidase; IL, interleukin; SIRT1, Silent mating type information regulation 2 homolog-1.

**Table 3:** Cardioactive property of ECH in selected models.

Models	Dosage	Activity/Mechanism	Refs
5-FU induced bone marrow depression mice	15 mg/kg for 12 days, p.o.	Stimulated the proliferation ability of bone marrow cells.	[82]
Bone marrow cells	0.1, 1, 10, 25 and 50 μM	Increased the number of total hematopoietic progenitor cells and granulocyte macrophage progenitor cells to healthy control mice level.	[82]
5-FU induced bone marrow depression mice	15 mg/kg/day for 12 days, p.o.	Improved the hematopoietic function of bone marrow, activated the PI3K signaling pathway.	[83]
Hypoxia-induced proliferation of rat pulmonary artery smooth muscle cells	0.35–0.4 mM of ECH	Stimulated the apoptosis of pulmonary artery smooth muscle cells, enhanced the protein and gene expression of caspase-3, Bax and Fas, decreased the expressions of Bcl-2 and hypoxia-inducible factor-1α.	[84]
TNF-α induced atherosclerosis of human umbilical vascular endothelial cells	40, 80 and 100 mg/L	Increased the survival of human umbilical vascular endothelial cells, reduced the secretion of lactate dehydrogenase, MDA, intercellular adhesion molecule-1 and the production of intracellular reactive oxygen.	[85]
Phenylephrine and KCl induced contracted of the isolated rat thoracic aortic ring	30–300 μM	Relaxed the endothelium-intact rings, enhanced the cyclic guanosine monophosphate production in aortic rings through NO-cyclic guanosine monophosphate pathway.	[86]
Noradrenaline induced contractions in isolated rat aortic strip	10–100 μM	Methanolic extract from the dried stems of *Cistanche tubulosa* inhibited the contractions, and ECH was responsible for this bioactive.	[87]

5-FU, 5-fluorouracil; PI3K, phosphatidylinositol 3-kinase.

**Table 4:** Anti-inflammatory property of ECH in selected models.

Models	Dosage/Concentration	Mechanism	Refs
H_2_O_2_ or pro-inflammatory cytokines induced injury on C3H/HeJ mice intestinal epithelial MODE-K cells	6.25–100 μg/mL	Stimulated cell proliferation, improved mucosal tissue repair, enhanced cell survival by reducing cell apoptosis, up-regulated TGF-β1 expression.	[91]
Lipopolysaccharide stimulated murine J774.1 cells, lipopolysaccharide/interferon-g stimulated mouse peritoneal exudate macrophages	2–200 μM	Inhibited and reduced nitrite accumulation and scavenged the nitrite generated from 1-propanamine-3-hydroxy-2-nitroso-1-propylhydrazino.	[92]
Dextran sulphate sodium-induced acute colitis in C57BL/6J mice, C3H/HeJ mice intestinal epithelial MODE-K cells	0.12–20 mg/kg/day for 7 days, p.o.	Suppressed the development of acute colitis, prevented colonic damage, protected intestinal epithelium from inflammatory injury, up-regulated the expression of TGF-β1, and increased the number of Ki67^+^ proliferating cells.	[88]
SD rats were abraded to generate erythema and cicatrization	0.4 mg/mL, topical	Decreased the edematous process, increased hyaluronan levels and less wound contraction.	[93]
Removed vocal fold lamina propria to generate injury in pigs	3–12 mg/mL for 15 days, topical	Improved the phonation threshold pressure and the vocal economy, maintained a stable hyaluronan and collagen content.	[94]
d-galactosamine/lipopolysaccharide-induced acute liver injury in mice and primary cultured mouse hepatocytes	25–100 mg/kg, p.o. 3–100 μg/mL	Inhibited the increase in aspartate aminotransaminase and alanine aminotransaminase, reduced the sensitivity of hepatocytes to TNF-α, inhibited the death of hepatocytes with IC_50_ was 10.2 μM.	[89]
d-galactosamine/lipopolysaccharide-induced acute liver injury in mice	60 mg/kg, p.o.	Improved the survival rate, attenuated acute hepatotoxicity, decreased alanine aminotransferase levels, improved histological signs, inhibited hepatocyte apoptosis, reduced myeloperoxidase, extracellular nucleosomes, high-mobility group box 1 and inflammatory cytokines.	[95]
CCl_4_-induced liver injury and oxidative stress in rats	50 mg/kg, i.p.	Reduced the serum ALT, AST, aspartate aminotransferase, capase-3 and TNF-α levels and hepatic MDA content as well as ROS production.	[90]
CCl_4_-induced liver injury and oxidative stress in rats	50 mg/kg, i.p.	Decreased ALT and AST levels, reduced the number of apoptotic hepatocytes and hepatic MDA content, increased hepatic SOD and GSH activities.	[96]
Human peripheral blood mononuclear cells	2–9 μg/mL	Increased cell proliferation and IL-10 content.	[97]

CCl_4_, carbon tetrachloride; TGF, transforming growth factor; TNF, tumor necrosis factor; ALT, alanine aminotransferase; AST, aspartate aminotransferase.

**Table 5:** Anti-oxidative property of ECH in selected models.

Models	Activity	Refs
DPPH radical scavenging activity	EC_50_ = 6.6 μM	[98]
ABTS radical cation assay	Scavenging capacity was ranged from 1.13% to 4.45% (% ascorbic acid by weight)	[99]
Hydroxyl radical generated by the xanthine/xanthine oxidase/Fe^2+^/EDTA system	Reaction between hydroxyl radical and ECH was 0.97 × 10^1^° L/mol/s.	[100]
Peroxynitrite radical scavenging activity	9.5-fold total oxidant scavenging capacity of Trolox	[101]
Superoxide anion (O^2−^.) radical scavenging activity generated by xanthine/xanthine oxidase	IC_50_ = 2.74 μM, stronger than αtocopherol	[105]
Inhibition of lipid peroxidation induced by ascorbic acid/Fe^2+^ and adenosine diphosphate/nicotinamide adenine dinucleotide phosphate/Fe^3+^	Stronger than αtocopherol or caffeic acid (*p* < 0.05)	[106]
Reduced the antioxidant response element of BACH1 in HaCaT cells	Enhanced heme oxygenase 1 mRNA levels by 40-fold in 72 h and cytoplasmic heme oxygenase 1 protein levels were also increased	[107]
Oxygen radicals (superoxide anion and hydroxyl radical), generated by the xanthine/xanthine oxidase/Fe^2+^/EDTA system, induced degradation of Type III collagen	IC_50_ = 15 μM	[108]
Oxygen free radicals generated by H_2_O_2_ induced damage in human dermal fibroblasts	IC_50_ = 3.17 μM	[109]
Cu^2+^-induced human LDL	IC_50_ = 1 μM	[110]
Briggs-Rauscher reaction method	Inhibition time was 350 s and concentration was 1.851 μM	[111]
Inhibition on the autoxidation of linoleic acid in CTAB micelles	IC_50_ = 10.9 μM	[103]
Inhibition of oxidative hemolysis in mouse erythrocytes	90% of Hemolysis inhibition at 3.0 μM within 3 h	[104]

ABTS, 2,2′-azino-bis3-ethylbenzthiazoline-6-sulphonic acid; CTAB, cetyl trimethylammonium bromide; EDTA, ethylene diamine tetraacetic acid; LDL, low-density lipoprotein.

**Table 6:** Anti-osteoporotic activity of ECH in selected models.

**Models**	**Dosage**	**Activity/Mechanism**	**Refs**
OVX rat model of osteoporosis	30, 90 and 270 mg/kg for 12 weeks, p.o.	Completely corrected the increased urine concentration of calcium, inorganic phosphorus, and hydroxyproline; enhanced bone quality, improved total bone mineral density and biomechanical strength of tibia, promoted the bone formation and suppressed the bone resorption.	[113]
OVX rat model of osteoporosis	30, 90 and 270 mg/kg/day for 12 weeks, p.o.	Improved total femur bone mineral density, bone microarchitecture and biomechanical properties, increased OPG level, decreased RANKL level; the anti-osteoporotic activity was similar to phytoestrogen but without influence the uterus and mammary gland.	[114]
Osteoblastic cells and MC3T3-E1 cells	0.01–100 nM	Stimulated the cell proliferation of osteoblast, induced expressions of BMP-2 and smad4 to activate BMP/smad pathway, promoted the phosphorylation of ERK1/2 to activate MAPK/ERK pathway.	[115]
Osteoblastic cells	5 × 10^−8^–5 × 10^−4^ mg/mL	Increased the expression of BMP-2 protein level.	[116]
Osteoblastic cells	5 × 10^−7^–5 × 10^−5^ mg/mL	Up-regulated the expression of OPN mRNA and protein of osteoblast.	[117]
Osteoblastic cells and MC3T3-E1 cells	0.01–10 nM	Increased cell proliferation, ALP activity, collagen I contents, osteocalcin levels, enhanced mineralization in osteoblasts and the ratio of OPG/RANKL.	[18]

OVX, ovariectomized; BMD, bone mineral density; BMP, bone morphogenetic proteins; ALP, alkaline phosphatase; OPN, osteopontin.

**Table 7:** Other activities of ECH in selected models.

**Other Bioactives**	**Models/Dosage**	**Activity/Mechanism**	**Refs**
Antidiabetic effect	Starch-loaded mice/125–500 mg/kg for 2 weeks, p.o.	Inhibited the rat lens aldose reductase with IC_50_ was 3.1 μM; inhibited the increase in postprandial blood glucose levels, improved glucose tolerance without producing significant changes in body weight or food intake.	[87]
Antiviral activity	Mouse macrophage model/100–1000 µg/mL	Possessed high antiviral activities with different antiviral profile and limited immune activation properties.	[118]
Anti-hepatic fibrosis effect	Hepatic stellate cell lines/125, 250 and 500 µg/mL	Inhibited hepatic stellate cell activation with IC_50_ was 520.3 µg/mL, suppressed the conduction of the signaling pathways in transforming growth factor–beta1/smad, including increasing the mRNA level and protein expression of smad7, and decreased both the mRNA and protein levels of smad2 and smad3 in hepatic stellate cell.	[119]
Anti-tumor activity	Pancreatic adenocarcinoma cell lines/20, 50, 100 µM	Inhibited the proliferation of pancreatic adenocarcinoma cells by inducing the production of reactive oxygen species and the perturbation of mitochondrial membrane potential and thus triggering apoptosis, and this activity was main through modulating MAPK activity.	[120]
Testis and sperm injury protect activity	Testicular and sperm toxicity induced by BPA/6 mg/kg for 6 weeks, p.o.	Reversed BPA-induced abnormality in sperm characteristics, testicular structure and normalized serum testosterone, enhanced the testosterone biosynthesis, increased expression of LDH-x, the key steroidogenic enzymes including StAR, CYP11A1, 3β-HSD, 17β-HSD and CYP17A1.	[121]

BPA, Bisphenol A; LDH: lactate dehydrogenase; StAR, steroidogenic acute regulatory protein; CYP11A1, cytochrome P450scc; CYP17A1, cytochrome P450 17A1; 3β-HSD, 3β-hydroxysteroid dehydrogenase/Δ5-Δ4isomerase; 17β-HSD, 17β-hydroxysteroid dehydrogenase.

## Abbreviations

The following abbreviations are used in this manuscript:

ABTS	2,2′-azino-bis 3-ethylbenzthiazoline-6-sulphonic acid
Aβ(25–35)	amyloid beta-protein fragment 25–35
AchE	acetylcholinesterase
AD	Alzheimer’s disease
ALP	alkaline phosphatase
ALT	alanine aminotransferase
Asp	aspartic acid
AST	aspartate aminotransferase
ATF3	activating transcription factors 3
BDNF	brain-derived neurotrophic factor
BMD	bone mineral density
BMP	bone morphogenetic proteins
BPA	bisphenol A
CCl_4_	carbon tetrachloride
ChAT	choline acetyltransferaxe
CHOP	C/EBP-homologous protein
CTAB	cetyl trimethylammonium bromide
CYP11A1	cytochrome P450scc
CYP17A1	cytochrome P450 17A1
DA	dopamine
DHBA	dihydroxybenzoic acid
DOPAC	3,4-dihydroxyphenyl acetic acid
DPPH	2,2-diphenyl-1-picrylhydrazylhydrate
ECH	echinacoside
EDTA	ethylene diamine tetraacetic acid
ERK	extracellular signal regulated kinase
5-FU	5-fluorouracil
GalN/LPS	d-galactosamine/lipopolysaccharide
GDNF	glial cell line-derived neurotrophic factor
Glu	glutamic acid
GSH	glutathione
GSH-Px	glutathione peroxidase
5-HT	5-hydroxytryptamine
HPTLC	high-performance thin-layer chromatography
HIAA	hydroxyindoleacetic acid
3β-HSD	3β-hydroxysteroid dehydrogenase/Δ5-Δ4 isomerase
17β-HSD	17β-hydroxysteroid dehydrogenase
HVA	homovanillic acid
HSCCC	high speed countercurrent Chromatography
iNOS	inducible NO synthase
IL	interleukin
LC	liquid chromatography
LDH	lactate dehydrogenase
LDL	low-density lipoprotein
LOD	limit of detection
MAPK	mitogen-activated protein kinase
MCAO	middle cerebral artery occlusion
MDA	malondialdehyde
MPP	1-methyl-4-phenylpyridinium
MPTP	1-methyl-4-phenyl-1,2,3,6-tetrahydropyridine
NE	norepinephrine
NF-kappa B	nuclear factor-kappa B
NO	nitric oxide
NOS	nitric oxide synthase
6-OHDA	6-hydroxydopamine
OPG	osteoprotegerin
OPN	osteopontin
OVX	ovariectomized
PD	Parkinson’s disease
PhGs	phenylethanoid glycosides
PI3K	phosphatidylinositol 3-kinase
RANKL	receptor activator for nuclear factor-κB ligand
ROS	reactive oxygen species
SCNA	synuclein alpha
SIRT1	silent mating type information regulation 2 homolog-1
SOD	superoxide dismutase
StAR	steroidogenic acute regulatory protein
TCM	traditional Chinese medicine
TGF	transforming growth factor
TNF	tumor necrosis factor
Trk	tropomyosin-related tyrosine kinase
OPG	osteoprotegerin
OPN	osteopontin
OVX	ovariectomized
PD	Parkinson’s disease
PhGs	phenylethanoid glycosides
PI3K	phosphatidylinositol 3-kinase
RANKL	receptor activator for nuclear factor-κB ligand
ROS	reactive oxygen species
SCNA	synuclein alpha
SIRT1	silent mating type information regulation 2 homolog-1
SOD	superoxide dismutase
StAR	steroidogenic acute regulatory protein
TCM	traditional Chinese medicine
TGF	transforming growth factor
TNF	tumor necrosis factor
Trk	tropomyosin-related tyrosine kinase

## References

[1] Alipieva K., Korkina L., Orhan I.E., Georgiev M.I. (2014). Verbascoside—A review of its occurrence, (bio)synthesis and pharmacological significance. Biotechnol. Adv..

[2] Stoll A., Renz J., Brack A. (1950). Isolation and constitution of echinacoside, a glycoside from the roots of *Echinacea angustifolia* DC. Helv. Chim. Acta.

[3] Kobayashi H., Komatsu J. (1983). Studies on the constituents of *Cistanchis* herba. 1. Yakugaku Zasshi.

[4] Ismail L.D., el-Azizi M.M., Khalifa T.I., Stermitz F.R. (1995). Verbascoside derivatives and iridoid glycosides from *Penstemon crandallii*. Phytochemistry.

[5] Zhang G.G., Yang Z.B., Wang Y., Yang W.R. (2013). Effects of *Astragalus membranaceus* root processed to different particle sizes on growth performance, antioxidant status, and serum metabolites of broiler chickens. Poult. Sci..

[6] Gousiadou C., Kokubun T., Martins J., Gotfredsen C.H., Jensen S.R. (2015). Iridoid glucosides in the endemic *Picconia azorica* (Oleaceae). Phytochemistry.

[7] Chen J., Cheng H., Zhang J., Zhang G., Ding W. (2003). Investigation on occurrence of *lycium barbarum* pests and its natural enemies at Ningxia. J. Chin. Med. Mater..

[8] Czerwinska M.E., Ziarek M., Bazylko A., Osinska E., Kiss A.K. (2015). Quantitative Determination of Secoiridoids and Phenylpropanoids in Different Extracts of *Ligustrum Vulgare* L. Leaves by a Validated HPTLC-Photodensitometry Method. Phytochem. Anal..

[9] Spanakis M., Niopas I. (2013). Determination of atenolol in human plasma by HPLC with fluorescence detection: Validation and application in a pharmacokinetic study. J. Chromatogr. Sci..

[10] Dong Y., Guo Q., Liu J., Ma X. (2018). Simultaneous determination of seven phenylethanoid glycosides in *Cistanches* Herba by a single marker using a new calculation of relative correction factor. J. Sep. Sci..

[11] Andary C., Tahrouch S., Marion C., Wylde R., Heitz A. (1992). Caffeic glycoside esters from *Jasminum nudiflorum* and some related species. Phytochemistry.

[12] Xie J., Tan F., Zhu J., Yue C., Li Q. (2012). Separation, purification and quantification of verbascoside from *Penstemon barbatus* (Cav.) Roth. Food Chem..

[13] Yang T.-X., Lu Y.-X., Guo Y.-H., Zhai Z.-X., Wang S.-A., Lu L.-Q., Yu G.-J. (2006). Stuided of dry matter accumulation and echinacoside ceontent of *Cistanche tubulosa* in Hubei plain. China J. Chin. Mater. Med..

[14] Xie J., Deng J., Tan F., Su J. (2010). Separation and purification of echinacoside from *Penstemon barbatus* (Can.) Roth by recycling high-speed counter-current chromatography. Life Sci..

[15] Perry N.B., Burgess E.J., Glennie V.L. (2001). Echinacea standardization: Analytical methods for phenolic compounds and typical levels in medicinal species. J. Agric. Food Chem..

[16] Xing Y.-X., Hu F.-Z., Dong Q., Peng M. (2012). Determination of echinacoside and acteoside in Tibetan herb *Lagotis brevituba* Maxim. Chin. J. Pharm. Anal..

[17] Yang T.X., Zhang X.H., Cai J.Z. (2007). Study on secondary metabolic organ of echinacoside in herbs of *Cistanche tubulosa*. China J. Chin. Mater. Med..

[18] Li F., Yang Y., Zhu P., Chen W., Qi D., Shi X., Zhang C., Yang Z., Li P. (2012). Echinacoside promotes bone regeneration by increasing OPG/RANKL ratio in MC3T3-E1 cells. Fitoterapia.

[19] Dong B., Yuan X., Zhao Q., Feng Q., Liu B., Guo Y., Zhao B. (2015). Ultrasound-assisted aqueous two-phase extraction of phenylethanoid glycosides from *Cistanche deserticola* Y. C. Ma stems. J. Sep. Sci..

[20] Cai H., Bao Z., Jiang Y., Wang X.Y., Fan X.T., Aierken M., Tu P.F. (2007). Study on processing method of *Cistanche tubulosa*. China J. Chin. Mater. Med..

[21] Li F., Yang X., Yang Y., Li P., Yang Z., Zhang C. (2015). Phospholipid complex as an approach for bioavailability enhancement of echinacoside. Drug Dev. Ind. Pharm..

[22] Lim E.K., Bowles D. (2012). Plant production systems for bioactive small molecules. Curr. Opin. Biotechnol..

[23] Zhu Y.H., Du F.L., Zhang S. (2003). Echinacoside determined in *Cistanche callus* using chlorogenic acid as a internal standard by HPLC. J. Hunan Univ. Chin. Med..

[24] Lu C.T., Mei X.G. (2003). Improvement of phenylethanoid glycosides production by a fungal elicitor in cell suspension culture of *Cistanche deserticola*. Biotechnol. Lett..

[25] Zhong L., Wu N.-Z. (2011). Effect of Echinacoside Content of *Cistanche deserticola* by Adding Precursors and Revulsant to Hosts. Biotechnology.

[26] Lv J.J., Hu G.S., Li J.K., Jia J.M. (2009). Effects of precursor feeding and fungal elicitors on secondary metabolits in cell suspension culture of *Cistanche deserticola*. J. Chin. Med. Mater..

[27] Xu L.S., Xue X.F., Fu C.X., Jin Z.P., Chen Y.Q., Zhao D.X. (2005). Effects of methyl jasmonate and salicylic acid on phenylethanoid glycosides synthesis in suspension cultures of *Cistanche deserticola*. Chin. J. Biotechnol..

[28] Mulani S.K., Guh J.H., Mong K.K. (2014). A general synthetic strategy and the anti-proliferation properties on prostate cancer cell lines for natural phenylethanoid glycosides. Org. Biomol. Chem..

[29] Martin K., Appel C. (2010). Polyphenols as dietary supplements: A double-edged sword. Nutr. Diet. Suppl..

[30] Del Rio D., Rodriguez-Mateos A., Spencer J.P., Tognolini M., Borges G., Crozier A. (2013). Dietary (poly) phenolics in human health: Structures, bioavailability, and evidence of protective effects against chronic diseases. Antioxid. Redox Signal..

[31] Jia C., Shi H., Wu X., Li Y., Chen J., Tu P. (2006). Determination of echinacoside in rat serum by reversed-phase high-performance liquid chromatography with ultraviolet detection and its application to pharmacokinetics and bioavailability. J. Chromatogr. B Anal. Technol. Biomed. Life Sci..

[32] Matthias A., Addison R., Penman K., Dickinson R., Bone K., Lehmann R. (2005). Echinacea alkylamide bioavailability and pharmacokinetics in humans after tablet ingestion. Life Sci..

[33] Ma Z.-G., Yang Z.-L., Feng Y.-P. (2008). Metabolites of Echinacoside in Rats Feces by HPLC-MS^n^. Chin. J. Nat. Med..

[34] Lei L., Song Z.H., Tu P.F., Li Y.Z., Wu L.J., Chen F.K. (2001). Metabolic regulation of phenylethanoid glycosides from Herba *cistanches* in dogs’ gastrointestine. Acta Pharm. Sin..

[35] Matthias A., Blanchfield J.T., Penman K.G., Toth I., Lang C.S., De Voss J.J., Lehmann R.P. (2004). Permeability studies of alkylamides and caffeic acid conjugates from *echinacea* using a Caco-2 cell monolayer model. J. Clin. Pharm. Ther..

[36] Matthias A., Penman K., Matovic N., Bone K., De Voss J., Lehmann R. (2005). Bioavailability of Echinacea constituents: Caco-2 monolayers and pharmacokinetics of the alkylamides and caffeic acid conjugates. Molecules.

[37] Gao Y., Zong C., Liu F., Fang L., Cai R., Shi Y., Chen X., Qi Y. (2015). Evaluation of the intestinal transport of a phenylethanoid glycoside-rich extract from *Cistanche deserticola* across the Caco-2 cell monolayer model. PLoS ONE.

[38] Wei L.-L., Chen H., Jiang Y., Tu P.-F., Zhong M., Liu F., Liu C.-Y. (2011). Determination of ECH on cerebral ischemia injury rat plasma and brain tissue by HPLC method. Chin. Pharmacol. Bull..

[39] Zhou J., Zeng P., Sun J.B., Wang F.Q., Zhang Q. (2013). Application of two-phase hollow fiber liquid phase microextraction coupled with high-performance liquid chromatography for the study of the echinacoside pharmacokinetics in Parkinson’s disease rat plasma. J. Pharm. Biomed. Anal..

[40] Lu H. (2005). Resolution of carotenoid isomers in *Lycium barbarum* L. by heuristic evolving latent projection. Chin. J. Chromatogr..

[41] Zhang W.-X., Ma J.-Y., Chen H., Jiang Y., Tu P.-F., Ding H. (2014). Effect of echinacoside on striatal and hippocampus extracellular fluid of monoamine neurotransmitter in Parkinson’s disease rats. Chin. Pharmacol. Bull..

[42] Chen H., Jing F.C., Li C.L., Tu P.F., Zheng Q.S., Wang Z.H. (2007). Echinacoside prevents the striatal extracellular levels of monoamine neurotransmitters from diminution in 6-hydroxydopamine lesion rats. J. Ethnopharmacol..

[43] Jing F.-C., Chen H., Li C.-L., Yan M.-Y. (2007). Effects of echinacoside on striatal extracellular levels of monomines neurotransmitters in 6-hydroxydopamine lesion rats. Chin. Pharmacol. Bull..

[44] Wang Y.H., Xuan Z.H., Tian S., Du G.H. (2015). Echinacoside Protects against 6-Hydroxydopamine-Induced Mitochondrial Dysfunction and Inflammatory Responses in PC12 Cells via Reducing ROS Production. Evid. Based Complement. Alternat. Med..

[45] Zhao Q., Gao J., Li W., Cai D. (2010). Neurotrophic and neurorescue effects of Echinacoside in the subacute MPTP mouse model of Parkinson’s disease. Brain Res..

[46] Zhao X., Pu X.-P., Geng X.-C. (2008). Effects of echinacoside on protein expression from substantia nigra and striatal tissue in mouse MPTP model of Parkinsons disease by using 2-dimensional electrophoresis analysis. Chin. Pharmacol. Bull..

[47] Zhang J., Zhang Z., Xiang J., Cai M., Yu Z., Li X., Wu T., Cai D. (2017). Neuroprotective Effects of Echinacoside on Regulating the Stress-Active p38MAPK and NF-kappaB p52 Signals in the Mice Model of Parkinson’s Disease. Neurochem. Res..

[48] Geng X., Tian X., Tu P., Pu X. (2007). Neuroprotective effects of echinacoside in the mouse MPTP model of Parkinson’s disease. Eur. J. Pharmacol..

[49] Zhao Q., Yang X., Cai D., Ye L., Hou Y., Zhang L., Cheng J., Shen Y., Wang K., Bai Y. (2016). Echinacoside Protects Against MPP(+)-Induced Neuronal Apoptosis via ROS/ATF3/CHOP Pathway Regulation. Neurosci. Bull..

[50] Wu C.R., Lin H.C., Su M.H. (2014). Reversal by aqueous extracts of *Cistanche tubulosa* from behavioral deficits in Alzheimer’s disease-like rat model: Relevance for amyloid deposition and central neurotransmitter function. BMC Complement. Altern. Med..

[51] Ding H., Chen H., Jiang Y., Tu P.-F., Ma J.-Y., Zhang W.-X. (2014). Effects of echinacoside on monoamine neurotransmitters in hippocampus and cortex of rats with Alzheimer’s disease. Chin. Pharmacol. Bull..

[52] Liu C.-L., Chen H., Jiang Y., Tu P.-F. (2013). Effects of echinacoside on behavior, oxygen free radical and cholinergic neurotransmitter metabolism rate of the rat model of vascular dementia. Chin. Pharmacol. Bull..

[53] Ding H., Chen H., Jiang Y., Tu P.-F., Ma J.-Y., Zhang W.-X. (2014). Effect of echinacoside on learning-memory ability and oxygen free radicals on model rats with Alzheimer’s disease. Chin. Pharmacol. Bull..

[54] Tian F., Zhang K., Kang A.-J., Jiang Y., Zhou S.-P., Zheng Z.-H. (2006). The Effect and Mechanism of Echinacoside on SAM-P/8’s Learning and Memory Ability. Lab. Anim. Sci. Manag..

[55] Liu C.-L., Chen H., Jiang Y., Tu P.-F., Zhong M., Ma J.-Y., Ding H., Zhang W.-X., Jin X.-M. (2013). Effects of echinacoside on extracellular acetylcholine and choline levels of hippocampus and striatum of cerebral ischemia rats. Acta Pharmacol. Sin..

[56] Zhang D., Li H., Wang J.B. (2015). Echinacoside inhibits amyloid fibrillization of HEWL and protects against Abeta-induced neurotoxicity. Int. J. Biol. Macromol..

[57] Qi X.-L., Xiao H.-T., Xiao Y., Hao X.-Y., Guang Z.-Z. (2011). Effects of Echinacoside and Isoacteoside on the Expression of Nicotinic Receptors in Neuroblastoma Cells. Lishizhen Med. Mater. Med. Res..

[58] Zhu M., Zhou M., Shi Y., Li W.W. (2012). Effects of echinacoside on MPP(+)-induced mitochondrial fragmentation, mitophagy and cell apoptosis in SH-SY5Y cells. Chin. J. Integr. Med..

[59] Deng M., Zhao J.Y., Tu P.F., Jiang Y., Chen J. (2005). Echinacoside rescues the SHSY5Y neruonal cells from TNFa-induced apoptosis. Chin. Pharmacol. Bull..

[60] Deng M., Zhao J.Y., Tu P.F., Jiang Y., Li Z.B., Wang Y.H. (2004). Echinacoside rescues the SHSY5Y neuronal cells from TNFalpha-induced apoptosis. Eur. J. Pharmacol..

[61] Shen L., Chen H., Zhu Q., Wang Y., Wang S., Qian J., Wang Y., Qu H. (2016). Identification of bioactive ingredients with immuno-enhancement and anti-oxidative effects from Fufang-Ejiao-Syrup by LC-MS(n) combined with bioassays. J. Pharm. Biomed. Anal..

[62] Kuang R., Sun Y., Yuan W., Lei L., Zheng X. (2009). Protective effects of echinacoside, one of the phenylethanoid glycosides, on H_2_O_2_-induced cytotoxicity in PC12 cells. Planta Med..

[63] Kuang R., Sun Y., Zheng X. (2010). Suppression of nitric oxide implicated in the protective effect of echinacoside on H_2_O_2_-induced PC12 cell injury. Nat. Prod. Commun..

[64] Kuang R., Sun Y.-G., Deng T.-L., Zheng X.-X. (2009). The protective effect and mechanisms of echinacoside on H_2_O_2_-injured PC12 cells. Chin. Pharmacol. Bull..

[65] Lu C.W., Lin T.Y., Huang S.K., Wang S.J. (2016). Echinacoside Inhibits Glutamate Release by Suppressing Voltage-Dependent Ca(2+) Entry and Protein Kinase C in Rat Cerebrocortical Nerve Terminals. Int. J. Mol. Sci..

[66] Zhong M., Liu C.-L., Chen H., Jiang Y., Tu P.F., Wei L.-L., Liu F. (2012). Effects of Echinacoside on Striatal Extracellular Levels of Hydroxyl Radical in Cerebral Ischemia Rats. Chin. Pharm. J..

[67] Zhong M., Chen H., Jiang Y., Tu P.F., Liu C.-L., Zhang W.-X., Ma J.-Y., Ding H. (2012). Effects of echinacoside on striatal extracellular levels of amino acid neurotransmitter in cerebral ischemia rats. Chin. Pharmacol. Bull..

[68] Zhong M., Chen H., Jiang Y., Tu P.F., Liu C.-L., Wei L.-L. (2012). Effects of echinacoside on monoamine neurotransmitters in bilateral brain tissue of rats with cerebral ischemia. Chin. J. New Drugs.

[69] Wei L.-L., Chen H., Jiang Y., Tu P.F., Du J., Zhong M., Liu F., Liu C.-L. (2011). Effects of echinacoside on Lipid Peroxidation in Cerebral Ischemia Rats. Chin. J. Inf. TCM.

[70] Du J., Chen H., Jiang Y., Tu P.F. (2010). Protective effect of echinacoside on cerebral ischemia rats. Lishizhen Med. Mater. Med. Res..

[71] Wei L.L., Chen H., Jiang Y., Tu P.F., Zhong M., Du J., Liu F., Wang L., Liu C.Y. (2012). Effects of echinacoside on histio-central levels of active mass in middle cerebral artery occlusion rats. Biomed. Environ. Sci..

[72] Wei L.L., Chen H., Jiang Y., Tu P.F., Zhong M., Du J., Liu F., Liu C.Y., Wang L. (2011). Effects of echinacoside on striatal extracellular levels of monoamine neurotransmitter in cerebral ischemia rats. Chin. Pharmacol. Bull..

[73] Feng X.Y., Zhu M., Zhang Q.Q., Chen Y.P., Li W.W. (2012). Selective protection of nigral dopaminergic neurons by echinacoside in a rat model of Parkinson disease induced by rotenone. J. Chin. Integr. Med..

[74] Zhu M., Lu C., Li W. (2013). Transient exposure to echinacoside is sufficient to activate Trk signaling and protect neuronal cells from rotenone. J. Neurochem..

[75] Ma J.-Y., Zhang W.-X., Chen H., Jiang Y., Tu P.F., Ding H. (2014). The protective effects of echinacoside on oxidative stress injury in vascular dementia rats. Chin. Pharmacol. Bull..

[76] Gulinuer M., Lei L., Tu P.F., Guo D., Lu J.-F. (2004). Study on Molecular Mechanism of Echinacoside for Against Aging. Acta Biochim. Biophys. Sin..

[77] Li Y., Song Y.-Y., Chu C.-M., Zhang H.-Q. (2011). Study on the Anti-aging Effect of Echinacoside. Chin. Pharm. J..

[78] Li Y., Song Y.-Y., Zhang H.-Q. (2010). Effect of Echinacoside on immune function and mitochondrial DNA relative content of aging mice. Chin. Pharmacol. Bull..

[79] Zhang A.-X., Lv W.-H., Xu S., Zhang H.-Q. (2009). Study on the anti-oxidation effect of echinacoside. Pract. Geriatr..

[80] Zhu H., Cheng C., Zhang C., Wang Z. (2011). Echinacoside suppresses cellular senscence of human fibroblastic cells by down-regulation of p53. J. Chin. Pharm. Sci..

[81] Xie H., Zhu H., Cheng C., Liang Y., Wang Z. (2009). Echinacoside retards cellular senescence of human fibroblastic cells MRC-5. Pharmazie.

[82] Lindner A., Santilli D., Hodgett J., Nerlinger C. (1960). Effects of 5-Fluorouracil on the Hematopoietic System of the Mouse. Cancer Res..

[83] Wang S., Zheng G., Tian S., Zhang Y., Shen L., Pak Y., Shen Y., Qian J. (2015). Echinacoside improves hematopoietic function in 5-FU-induced myelosuppression mice. Life Sci..

[84] Gai X.Y., Tang F., Ma J., Zeng K.W., Wang S.L., Wang Y.P., Wuren T.N., Lu D.X., Zhou Y., Ge R.L. (2014). Antiproliferative effect of echinacoside on rat pulmonary artery smooth muscle cells under hypoxia. J. Pharmacol. Sci..

[85] Li H., Song A.-Q., Xue J.-H., Zhou Y.-H. (2013). Protective effect of echinacoside on vascular endothelial cells. J. Xi’an Jiaotong Univ. Med. Sci..

[86] He W.J., Fang T.H., Ma X., Zhang K., Ma Z.Z., Tu P.F. (2009). Echinacoside elicits endothelium-dependent relaxation in rat aortic rings via an NO-cGMP pathway. Planta Med..

[87] Morikawa T., Ninomiya K., Imamura M., Akaki J., Fujikura S., Pan Y., Yuan D., Yoshikawa M., Jia X., Li Z. (2014). Acylated phenylethanoid glycosides, echinacoside and acteoside from *Cistanche tubulosa*, improve glucose tolerance in mice. J. Nat. Med..

[88] Jia Y., Guan Q., Jiang Y., Salh B., Guo Y., Tu P., Du C. (2014). Amelioration of Dextran Sulphate Sodium-Induced Colitis in Mice by Echinacoside-Enriched Extract of *Cistanche tubulosa*. Phytother. Res..

[89] Morikawa T., Pan Y., Ninomiya K., Imura K., Matsuda H., Yoshikawa M., Yuan D., Muraoka O. (2010). Acylated phenylethanoid oligoglycosides with hepatoprotective activity from the desert plant *Cistanche tubulosa*. Bioorg. Med. Chem..

[90] Wu Y., Xu G.-L., Lou M., Zeng Z. (2008). The protective effect of echinacoside on acute liver injury in rats. Chin. J. Gastroenterol. Hepatol..

[91] Jia Y., Guan Q., Guo Y., Du C. (2012). Echinacoside stimulates cell proliferation and prevents cell apoptosis in intestinal epithelial MODE-K cells by up-regulation of transforming growth factor-beta1 expression. J. Pharmacol. Sci..

[92] Xiong Q., Tezuka Y., Kaneko T., Li H., Tran L.Q., Hase K., Namba T., Kadota S. (2000). Inhibition of nitric oxide by phenylethanoids in activated macrophages. Eur. J. Pharmacol..

[93] Speroni E., Govoni P., Guizzardi S., Renzulli C., Guerra M.C. (2002). Anti-inflammatory and cicatrizing activity of *Echinacea pallida* Nutt. root extract. J. Ethnopharmacol..

[94] Rousseau B., Tateya I., Lim X., Munoz-del-Rio A., Bless D.M. (2006). Investigation of anti-hyaluronidase treatment on vocal fold wound healing. J. Voice.

[95] Li X., Gou C., Yang H., Qiu J., Gu T., Wen T. (2014). Echinacoside ameliorates d-galactosamine plus lipopolysaccharide-induced acute liver injury in mice via inhibition of apoptosis and inflammation. Scand. J. Gastroenterol..

[96] Wu Y., Li L., Wen T., Li Y.Q. (2007). Protective effects of echinacoside on carbon tetrachloride-induced hepatotoxicity in rats. Toxicology.

[97] Senchina D.S., Strauch J.H., Hoffmann G.B., Shah N.B., Laflen B.K., Dumke B.L., Dao C.T., Dias A.S., Perera M.A. (2011). Phytochemical and immunomodulatory properties of an *Echinacea laevigata* (Asteraceae) tincture. J. Altern. Complement. Med..

[98] Pellati F., Benvenuti S., Magro L., Melegari M., Soragni F. (2004). Analysis of phenolic compounds and radical scavenging activity of *Echinacea* spp.. J. Pharm. Biomed. Anal..

[99] Sloley B.D., Urichuk L.J., Tywin C., Coutts R.T., Pang P.K., Shan J.J. (2001). Comparison of chemical components and antioxidants capacity of different *Echinacea* species. J. Pharm. Pharmacol..

[100] Wang P., Zheng R., Gao J., Jia Z., Wang W., Yao S., Zhang J., Lin N. (1996). Reaction of hydroxyl radical with phenylpropanoid glycosides from *Pedicularis* species: A pulse radiolysis study. Chin. Acad. Sci..

[101] Tai B.H., Jung B.Y., Cuong N.M., Linh P.T., Tung N.H., Nhiem N.X., Huong T.T., Anh N.T., Kim J.A., Kim S.K. (2009). Total peroxynitrite scavenging capacity of phenylethanoid and flavonoid glycosides from the flowers of *Buddleja officinalis*. Biol. Pharm. Bull..

[102] Hu G.S., Hur Y.J., Jia J.M., Lee J.H., Chung Y.S., Yi Y.B., Yun D.J., Park S.K., Kim D.H. (2011). Effects of 2-aminoindan-2-phosphonic acid treatment on the accumulation of salidroside and four phenylethanoid glycosides in suspension cell culture of *Cistanche deserticola*. Plant Cell Rep..

[103] Zheng R.L., Wang P.F., Li J., Liu Z.M., Jia Z.J. (1993). Inhibition of the autoxidation of linoleic acid by phenylpropanoid glycosides from *Pedicularis* in micelles. Chem. Phys. Lipids.

[104] Li J., Wang P.F., Zheng R., Liu Z.M., Jia Z. (1993). Protection of phenylpropanoid glycosides from *Pedicularis* against oxidative hemolysis in vitro. Planta Med..

[105] Mucaji P., Zahradnikova A., Bezakova L., Cupakova M., Rauova D., Nagy M. (2011). HPLC determination of antilipoxygenase activity of a water infusion of *Ligustrum vulgare* L. leaves and some of its constituents. Molecules.

[106] Xiong Q., Kadota S., Tani T., Namba T. (1996). Antioxidative effects of phenylethanoids from *Cistanche deserticola*. Biol. Pharm. Bull..

[107] Sgarbossa A., Dal Bosco M., Pressi G., Cuzzocrea S., Dal Toso R., Menegazzi M. (2012). Phenylpropanoid glycosides from plant cell cultures induce heme oxygenase 1 gene expression in a human keratinocyte cell line by affecting the balance of NRF2 and BACH1 transcription factors. Chem. Biol. Interact..

[108] Facino R.M., Carini M., Aldini G., Saibene L., Pietta P., Mauri P. (1995). Echinacoside and caffeoyl conjugates protect collagen from free radical-induced degradation: A potential use of *Echinacea* extracts in the prevention of skin photodamage. Planta Med..

[109] Mensah A.Y., Sampson J., Houghton P.J., Hylands P.J., Westbrook J., Dunn M., Hughes M.A., Cherry G.W. (2001). Effects of Buddleja globosa leaf and its constituents relevant to wound healing. J. Ethnopharmacol..

[110] Dalby-Brown L., Barsett H., Landbo A.K., Meyer A.S., Molgaard P. (2005). Synergistic antioxidative effects of alkamides, caffeic acid derivatives, and polysaccharide fractions from *Echinacea purpurea* on in vitro oxidation of human low-density lipoproteins. J. Agric. Food Chem..

[111] Cervellati R., Renzulli C., Guerra M.C., Speroni E. (2002). Evaluation of antioxidant activity of some natural polyphenolic compounds using the Briggs-Rauscher reaction method. J. Agric. Food Chem..

[112] Wang T., Zhang X., Xie W. (2012). *Cistanche deserticola* YC Ma, “Desert ginseng”: A review. Am. J. Chin. Med..

[113] Li F., Yang X., Yang Y., Guo C., Zhang C., Yang Z., Li P. (2013). Antiosteoporotic activity of echinacoside in ovariectomized rats. Phytomedicine.

[114] Yang X., Li F., Yang Y., Shen J., Zou R., Zhu P., Zhang C., Yang Z., Li P. (2013). Efficacy and safety of echinacoside in a rat osteopenia model. Evid. Based Complement. Alternat. Med..

[115] Fang H.-L., Li J.-X., Yao L.-M., Tao Z. (2015). Echinacoside promotes cell proliferation of rat osteoblast through activating of ERK/BMP-2 signaling pathway. Med. Forum.

[116] Xing X.X., Liu Z.-J., Han B. (2011). Effects of Acteoside and Echinacoside on the Expression of the BMP2 in Rat Osteoblast. Prog. Vet. Med..

[117] Li C.-H., Liu Z.-J., Zheng S.-J., Han B., Wang J.-F. (2013). Effect of Echinacoside on the Expression of the OPN mRNA and Protein in Rat Osteoblasts in vitro. Chin. Anim. Husb. Vet. Med..

[118] Vohra S., Adams D., Hudson J.B., Moore J.A., Vimalanathan S., Sharma M., Burt A.J., Lamont E., Lacaze N., Arnason J.T. (2009). Selection of natural health products for clinical trials: A preclinical template. Can. J. Physiol. Pharmacol..

[119] You S.P., Ma L., Zhao J., Zhang S.L., Liu T. (2016). Phenylethanol Glycosides from *Cistanche tubulosa* Suppress Hepatic Stellate Cell Activation and Block the Conduction of Signaling Pathways in TGF-beta1/smad as Potential Anti-Hepatic Fibrosis Agents. Molecules.

[120] Wang W., Luo J., Liang Y., Li X. (2016). Echinacoside suppresses pancreatic adenocarcinoma cell growth by inducing apoptosis via the mitogen-activated protein kinase pathway. Mol. Med. Rep..

[121] Jiang Z., Wang J., Li X., Zhang X. (2016). Echinacoside and *Cistanche tubulosa* (Schenk) R. wight ameliorate bisphenol A-induced testicular and sperm damage in rats through gonad axis regulated steroidogenic enzymes. J. Ethnopharmacol..

